# Pachychoroid neovasculopathy has clinical properties that differ from conventional neovascular age-related macular degeneration

**DOI:** 10.1038/s41598-023-33936-z

**Published:** 2023-05-06

**Authors:** Ai Kuranami, Ruka Maruko, Ichiro Maruko, Taiji Hasegawa, Tomohiro Iida

**Affiliations:** Department of Ophthalmology, Tokyo Women’s Medical University, 8-1 Kawadacho, Shinjuku-Ku, Tokyo, 162-8666 Japan

**Keywords:** Medical research, Outcomes research

## Abstract

To determine the clinical properties of pachychoroid neovasculopathy (PNV) that differ from conventional neovascular age-related macular degeneration (nAMD) and suggest that they are different clinical entities. To accomplish this, we reviewed the medical records of 100 consecutive patients diagnosed with nAMD. All of the patients were Japanese, and their mean age was 75.5 years. There were 72 men and 28 women. For the bilateral cases, only the right eye was analyzed. An eye was diagnosed with PNV when a macular neovascularization (MNV) was detected just above the dilated choroidal vessels. The Indocyanine green angiographic (ICGA) and *en face* optical coherence tomographic (OCT) images were used to assess the vertical symmetry of the medium and large choroidal vessels. The subfoveal choroidal thickness (SCT) was also measured manually in the OCT images. After reclassification, there were 29 (29%) patients with typical nAMD (25 with type 1 MNV, 4 with type 2 MNV), 43 (43%) with PNV, 21 (21%) with polypoidal choroidal vasculopathy, and 7 (7%) with retinal angiomatous proliferation. Of the 43 PNV, 17 (39.5%) had polypoidal lesions and 26 (60.5%) had no polypoidal lesions. The percentage of eyes with vertical asymmetry of the medium and large choroidal vessels was significantly greater in the 35 PNV (81.4%) than in the 16 non-PNV (28.1%; *P* < 0.01) cases. The mean SCT was significantly thicker in the PNV eyes than in the non-PNV eyes (298 ± 96 μm vs. 228 ± 82 μm; *P* < 0.01). The response of PNV to anti-vascular endothelial growth factor treatments was better than that of non-PNV eyes [higher dry macula rate after the loading period (90.9% vs. 59.1%), fewer total number of injections (11.0 ± 2.9 vs. 13.4 ± 3.2), and longer treatment intervals for the anti-VEGF therapy (8.4 ± 3.1 vs. 13.4 ± 3.2 weeks) at 2 years (all *P *< 0.01)]. These differences in the morphology and response to anti-VEGF treatments suggest that PNV is a separate clinical entity to conventional nAMD.

## Introduction

Macular neovascularization (MNV) secondary to age-related macular degeneration (AMD) was previously classified as typical neovascular AMD (nAMD), polypoidal choroidal vasculopathy (PCV), or retinal angiomatous proliferation (RAP). Now they are classified as type 1 MNV, type 2 MNV, type 3 MNV, and PCV as subtypes of type 1 MNV^1^. An earlier study that examined the percentage of each subtype of MNV secondary to AMD in the Japanese population reported that the type 1 and type 2 MNVs accounted for 45%, PCV accounted for another 45%, and type 3 MNV (RAP) accounted for about 6%^2,3^. The proportion of the PCV and type 3 MNV (RAP) types in the Japanese population differed significantly from that in Western countries, and the MNV secondary to AMD patients in Asia had fewer drusen-associated disease and more with prior central serous chorioretinopathy than in the Western countries^4^.

In 2013, Freund et al. proposed a new disease entity called pachychoroid neovasculopathy (PNV)^4,5^. The pathogenesis of PNV was not associated with the presence of drusen, and it was believed that PNV was associated with the medium and large sized choroidal vessels in Haller’s layer. It was suggested that the pachyvessels thickened the choroid which then compressed the vessels in the inner choroidal layer. Although the presence of PNV may explain the MNV in the Japanese population, this definition of PNV has not been fully accepted^6^. The PNV cases were still included in those that were treated as MNV secondary to AMD. Some clinicians believe that PCV is part of PNV, and these conflicting diagnosis of MNV has led to some confusion in identifying and treating eyes with MNV secondary to AMD^7–10^.

The purpose of this study was to determine whether cases diagnosed as MNV secondary to treatment-naïve AMD can be reclassified into different clinical entities. To accomplish this, we reviewed the medical records of 100 patients who were newly diagnosed by conventional criteria as nAMD. We shall show that many of these nAMD eyes had clinical findings that were distinct from that of other eyes diagnosed as nAMD. Thus, they should be classified as a separate clinical entity.

## Results

**Figure 1:**
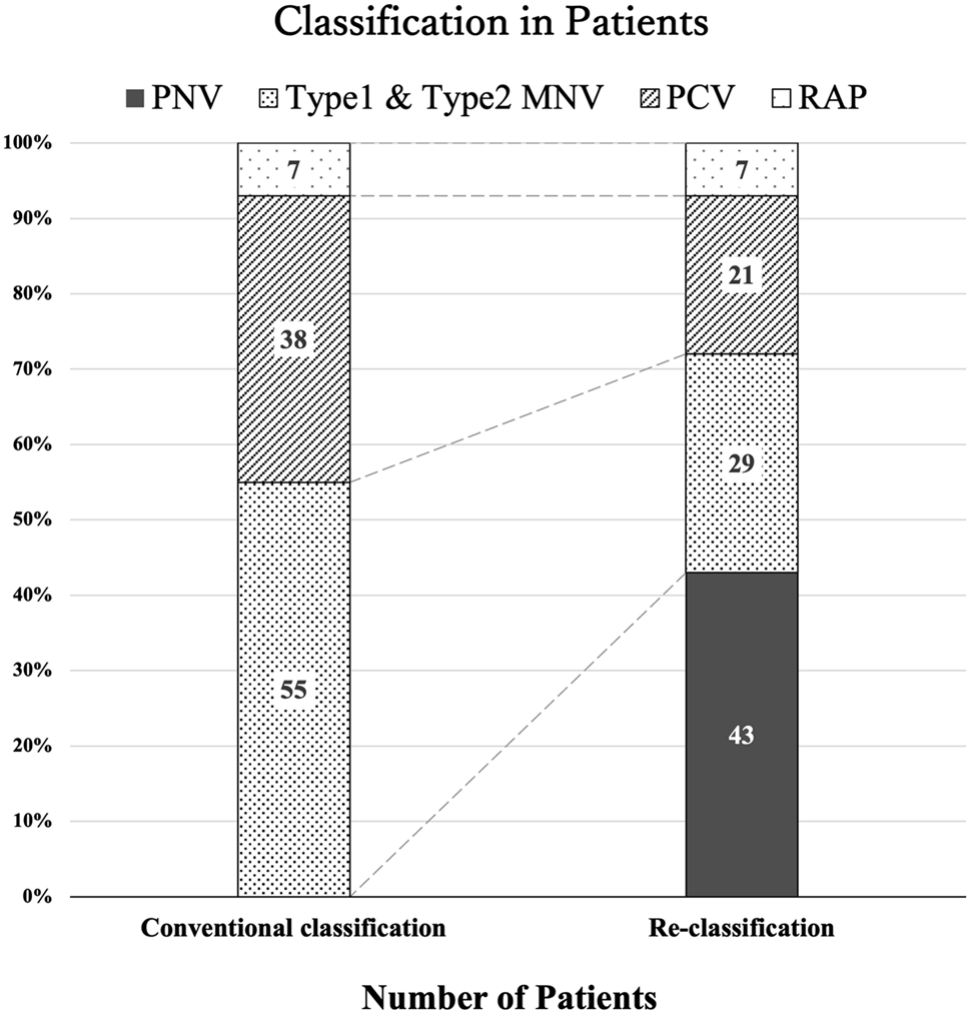
Conventional classification and re-classification of patients with MNV secondary to age-related macular degeneration and including PNV. *PNV* pachychoroid neovasculopathy, *MNV* macular neovascularization, *PCV* polypoidal choroidal vasculopathy, *RAP* retinal angiomatous proliferation

Of 100 patients, 55 (55%) were diagnosed with typical neovascular AMD (45 type 1 MNV, 10 type 2 MNV), 38 (38%) with PCV, and 7 (7%) with type 3 MNV according to the conventional classification (Fig. 1. Left). After re-diagnosing with the inclusion of PNV, the re-classification was 43 (43%) with PNV and 57 (57%) with MNV secondary to AMD as non-PNV group. The non-PNV group included 29 with typical neovascular AMD (25 type 1 MNV, 4 type 2 MNV), 21 with PCV, and 7 with RAP. (Fig. 1, right) The classification of 111 eyes including 11 bilateral cases is presented in Supplementary Fig S1. Seven of the 43 PNV eyes had a subfoveal choroidal thickness (SCT) < 200 µm.

**Figure 2:**
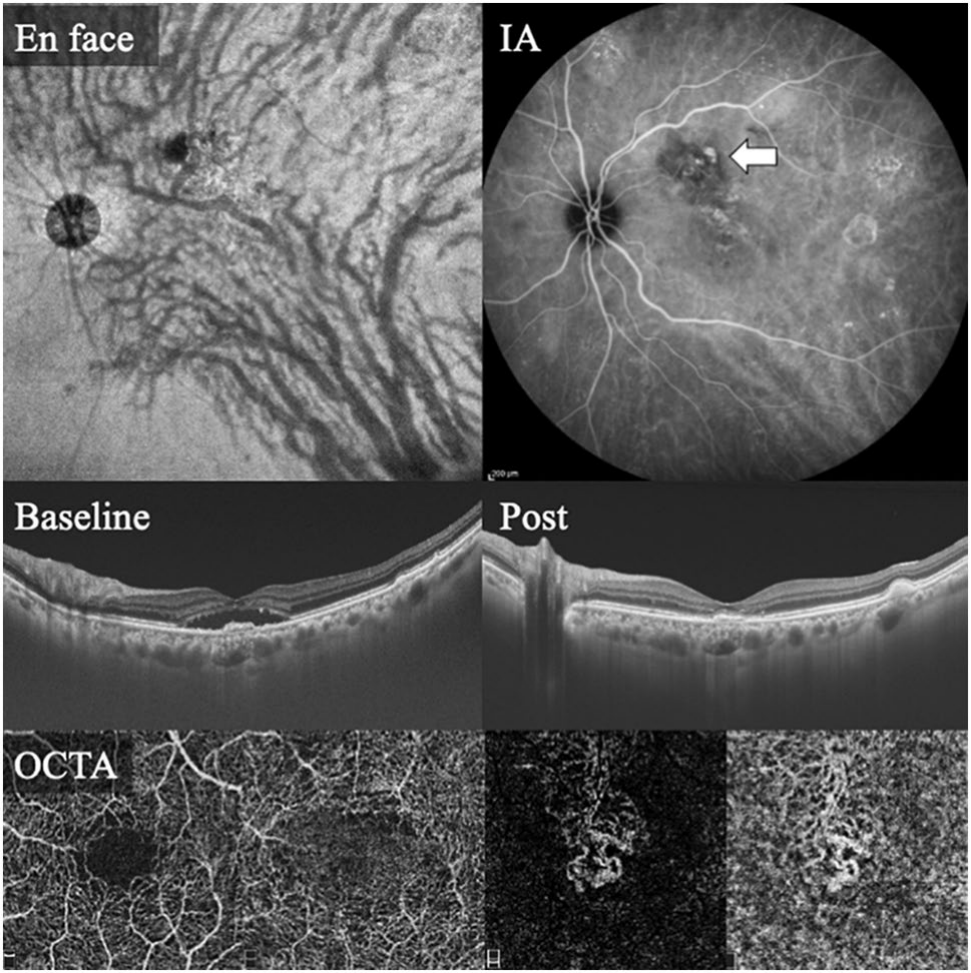
Findings in an 81-year-old man with PCV by conventional classification or with PNV after re-classification. *En face* OCT shows vertical asymmetry of the choroidal vessels with dilated choroidal vessels (pachyvessels) corresponding to the polypoidal lesions in the ICGA images. ICGA shows an abnormal network of vessels connecting superiorly from the central fovea with a polypoidal lesion at its apical tip (white arrow). Baseline OCT shows SRF with elevated RPE. The SCT is 385 µm. OCTA shows an abnormal network of vessels including those in the central fovea as MNV. *OCT* optical coherence tomography, *ICGA* indocyanine green angiography, *SRF* subretinal fluid, *RPE* retinal pigment epithelium, *SCT* subfoveal choroidal thickness, *OCTA* optical coherence tomography angiography, *MNV* macular neovascularization, *PCV* polypoidal choroidal vasculopathy.

**Figure 3:**
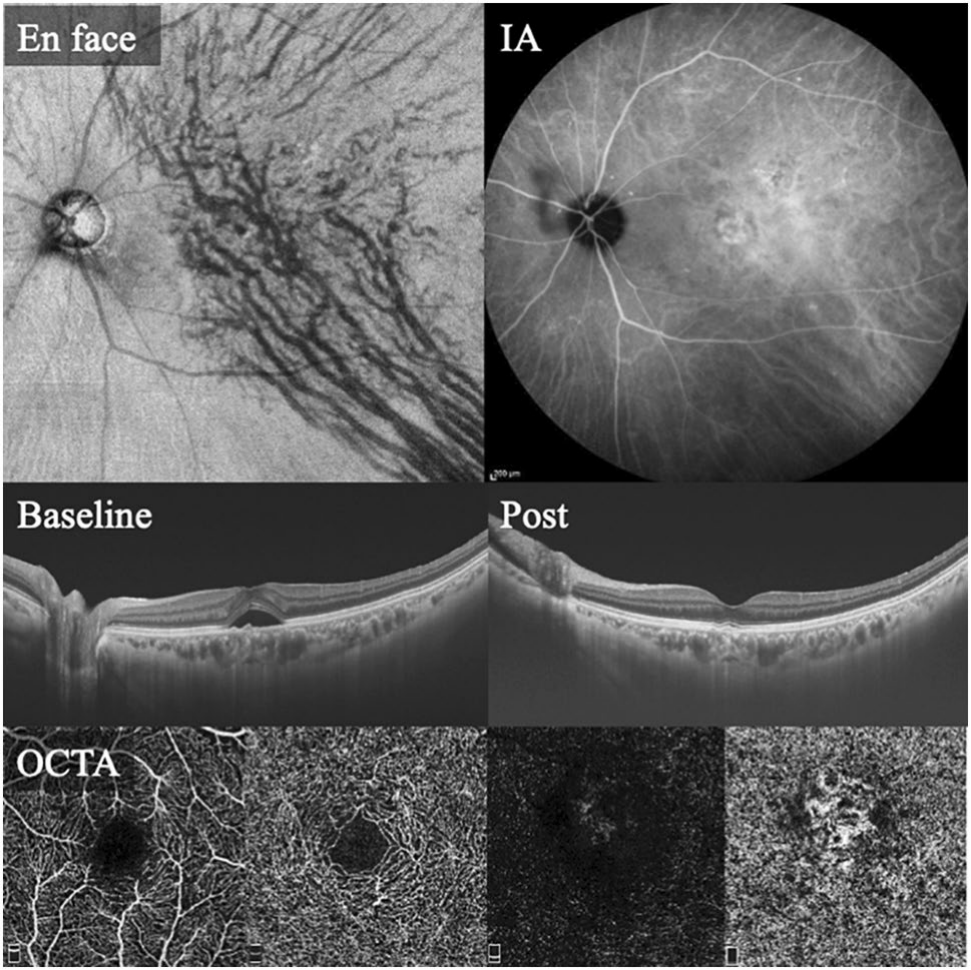
Findings in a 70-year-old woman. A representative case of a type 1 MNV with conventional classification or a PNV with reclassification. *En face* OCT image shows vertical asymmetry of choroidal vessels, with dilated choroidal vessels (pachyvessels) around the fovea. ICGA shows no polypoidal lesion. Baseline OCT shows SRF with elevated RPE; SCT is 317 µm. OCTA shows MNV at the fovea. *OCT* optical coherence tomography, *IA* indocyanine green angiography, *SRF* subretinal fluid, *RPE* retinal pigment epithelium, *SCT* subfoveal choroidal thickness, *OCTA* optical coherence tomography angiography, *MNV* macular neovascularization, *PNV* pachychoroid neovasculopathy

Of the 43 eyes with PNV, 17 (39.5%) had polypoidal lesions and 26 (60.5%) had no polypoidal lesions. Images of representative cases of PNV are shown in Figs. 2 and 3. The characteristics of the PNV group and non-PNV group are shown in Table 1. The mean SCT was significantly thicker in the PNV group (298 ± 96 µm) than in the non-PNV group (228 ± 82 µm; *P* < 0.01). The percentage of eyes with a vertical asymmetry of the medium and large choroidal vessels was significantly greater in the 35 PNV (81.4%) than in 18 non-PNV (31.6%; *P* < 0.01). The percentage of patients with drusen was 19.3% in the non-PNV group. Drusen were not observed in the PNV group.

**Table 1:** Characteristics of patients with macular neovascularization in PNV and non-PNV group.

	Non PNV	PNV	*P*-value
Patients, n	57	43	
Eye, n	57	43	
Age (years)	76.5 ± 8.5	74.2 ± 9.2	0.263*
Sex, n (%) Men	39 (68.4)	32 (74.4)	0.657†
SCT (µm)	228 ± 82	298 ± 96	< 0.01*
Vertical asymmetry of choroidal vessels, n (%)	18 (31.6)	35 (81.4)	< 0.01†
Drusen, n (%)	11 (19.3%)	0 (0%)	< 0.01†

*PNV* Pachychoroid neovasculopathy, *SCT* Subfoveal choroidal thickness.

*Mann–Whitney U test. †A chi-square tests.

Twenty-two PNV and 22 non-PNV patients were followed for at least 2 years after beginning the anti-vascular endothelial growth factor (anti-VEGF) treatment with the treat and extend (TAE) regimen (Table 2). RAP was not included in the cases evaluated for treatment responses. The percentage of eyes with a dry macula was significantly higher in the PNV eyes (20 eyes, 90.9%) than in the non-PNV (13 eyes, 59.1%) after the loading phase of one of three anti-VEGF drugs (*P *< 0.01). The mean number of injections at 2 years after the start of treatment was 11.0 ± 2.9 in the PNV and 13.4 ± 3.2 in non-PNV groups, and the dosing interval was 12.1 ± 2.9 and 8.4 ± 3.1 weeks, respectively. These findings indicated that fewer doses and longer intervals between anti-VEGF injections were used in the PNV group (both *P* < 0.01).

**Table 2:** Treatment response after 2 years in PNV and non-PNV group.

	Non-PNV (n = 22)	PNV (n = 22)	*P*-value
Sex, n (%) Men	16 (72.7)	17 (77.2)	1†
Age (years)	77.7 ± 6.3	74.7 ± 7.0	0.21*
MNV type, n (%)			
PCV	9	9	0.24†
Type 1	12	8	
Type 2	1	5	
Anti-VEGF			
Aflibercept	22	21	1†
Ranibizumab		1	
Dry macula after loading phase	13 (59.1%)	20 (90.9%)	< 0.03†
Number of injections	13.4 ± 3.2	11.0 ± 2.9	< 0.01*
*Interval at*			
2 years (month)	8.4 ± 3.1	12.1 ± 2.9	< 0.01*
SCT (µm)	231 ± 80	296 ± 89	< 0.02*
1 year	166 ± 82	230 ± 74	< 0.02*
2 years	160 ± 70	216 ± 71	< 0.02*

*PNV* Pachychoroid neovasculopathy, *MNV* macular neovascularization, *SCT* subfoveal choroidal thickness.

*Mann–Whitney U test. †A chi-square tests.

The SCT was 296 ± 89 μm in the PNV group and 231 ± 80 μm in the non-PNV group prior to the anti-VEGF treatment. The SCT was significantly reduced immediately after the loading phase at 1 year and also at 2 years after the anti-VEGF treatment (all *P* < 0.02).

For the 56 patients not included in the treatment analysis, there were 6 cases of pro re nata regimen, 9 cases of photodynamic therapy, 2 cases of no treatment, and 39 cases of lost to transfer to other medical institutions or dropout.

## Discussion

Our results showed that 47.3% of the of the conventionally diagnosed neovascular AMD (type 1 and type 2 MNV) and 44.7% of the PCV were re-classified as PNV. In the end, 43% of all conventionally diagnosed nAMD cases were classified as PNV. These results indicate that PNV is most common in elderly patients with MNV, and nearly one-half of the patients treated as type 1 or type 2 MNV or PCV in the clinic probably should be diagnosed as being PNV.

An earlier study by Miyake et al.^11^ reported that the percentage of eyes with PNV in eyes diagnosed as nAMD was 19.5% (39/200), and Borreli et al.^12^ reported that 25.2% (35/139) of nAMD eyes were PNV eyes. The diagnosis of PNV in previous studies were made by the presence of: (1) a subfoveal choroidal thickness of ≥ 200 μm, (2) no drusen or the absence of hard drusen, (3) choroidal vascular hyperpermeability, (4) RPE abnormality independent of any MNV lesions, (5) presence of dilated choroidal vessels or thickening below the type 1 MNV, and (6) a history of CSC. The major difference between the criteria for PNV of these earlier studies and ours was that the SCT must be ≥ 200 μm which was not a criterion for our study. However, the definitions of pachychoroid and PNV are not definitively set^6^, and our classification is just one set of signs.

It was recently suggested that the mechanism causing the development of PNV is the dilated choroidal vessels or pachyvessels that compress the choroidal capillaries immediately above them resulting in ischemia. This then leads to the appearance of a MNV^13,14^. On the other hand, it is known that the gradual thinning of the choroid is inversely correlated with aging^15–17^. Therefore, if an absolute choroidal thickness value in a normal eye is set at > 200 µm, the cases with a thin choroid would be excluded from the classification of PNV. We did not use the choroidal thickness as a criterion for PNV which may be the reason why we had a higher percentage of PNV than reported. In fact, there were 7 of 43 eyes with a SCT < 200 µm that were judged to have PNV.

The choroidal arteries pass into the eye as the short posterior ciliary arteries and the blood drains out of the eye through the vortex veins. It has been reported that the choroidal vessels in pachychoroid spectrum diseases have a vertical asymmetry due to a congestion of the vortex veins^18–20^. In our study, as in these previous studies, 35 of 43 eyes (81.4%) with PNV had a high degree of vertical asymmetry of the choroidal veins which was significantly higher than the 18 of 57 eyes (31.6%) with non-PNV other than PNV.

The eyes with PNV had a better response to treatment with anti-VEGF agents than non-PNV eyes. They required fewer anti-VEGF injections and had greater benefits of PDT^21–30^. In our cohort, as in previous studies, the PNV patients had a higher percentage of dry macula after the loading period, fewer total number of injections, and longer treatment intervals for the anti-VEGF therapy. Some studies have reported that the intraocular cytokine levels in PNV eyes were different from that in non-PNV eyes^31–33^, and it is possible that PNV has a better response to anti-VEGF therapy than eyes with non-PNV because of the different contributions of VEGF to their pathologies.

**Figure 4:**
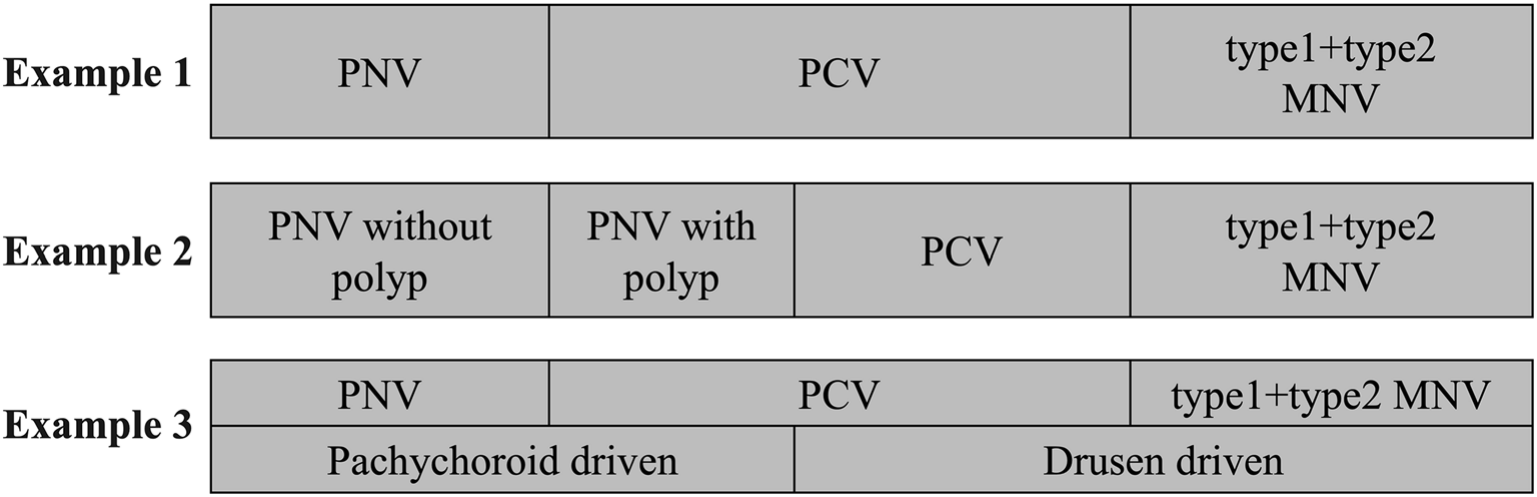
Different ways to divide the MNV including PNV. Example 1. PNV, PCV, and type 1/type 2 MNV. Example 2. PNV is divided by the presence or absence of polypoidal lesions. Example 3. PNV, PCV, and type 1/type 2 MNV are divided into pachychoroid-driven and drusen-driven. *MNV* macular neovascularization, *PNV* pachychoroid neovasculopathy, *PCV* polypoidal choroidal vasculopathy.

Proposals for the reclassification of MNV secondary to AMD have been ongoing since PNV was first proposed as a separate clinical entity with an absence of drusen formation for its pathogenesis^34^. Because PNV was also found to be independent of the presence or absence of polypoidal lesions, there is currently discussions on how to separate PNV from PCV. For example, as shown in Fig. 4, there are various ways to divide PNV, PCV, and type 1 and type 2 MNV, and also on how to classify PNV by the presence or absence of polypoidal lesions. Eyes with PCV included those related to pachychoroid and AMD of drusen origin^35,36^. Yamashiro et al.^35^ reported that both PCV and type 1 and type 2 MNVs can be divided into pachychoroid-driven and drusen-driven. However, in our cohort, only 19.3% of the non-PNV patients had drusen. This suggested that there may be a mechanism for the development of MNVs that is neither pachychoroid-driven nor drusen-driven. However, this is unknown at this time and will be an issue for further investigations. The classification of MNV secondary to AMD should be based on the presence of polypoidal lesions, drusen, genetic background, and response to treatment.

There are limitations in this study. First, this was a retrospective study with its inherent biases. Second, the classifications were performed at a single institution. Nevertheless, we believe that this is a meaningful study because of the relatively large number of cases and the reference to the response to treatment.

In conclusion, we reclassified conventional MNV cases secondary to AMD and found that eyes with PNV were the most common type of MNV in elderly patients. This indicates that nearly one-half of the patients diagnosed and treated as type 1 or type 2 MNV or PCV in the clinical practice may be PNV. The results also showed that PNV eyes respond better to anti-VEGF treatment, and thus it is important to classify and treat PNV separately from conventional diagnosis.

## Methods

The procedures used in this study conformed to the tenets of the Declaration of Helsinki and were approved by the Institutional Review Board of Tokyo Women’s Medical University. All patients signed a written informed consent form before beginning the treatments.

We identified 111 eyes of 100 consecutive patients (79 eyes of 72 men and 32 eyes of 28 women) newly diagnosed with MNV secondary to treatment-naïve AMD between 2013 and 2021 at the Department of Ophthalmology, Tokyo Women's Medical University Hospital. No new AMD cases were excluded from the study except those for which imaging was not available. All patients were Japanese. For the 11 bilateral cases (11%), only the right eye was used in the statistical analysis. The mean age was 75.6 ± 8.84 years. All patients underwent fundus examination by slit-lamp microscopy, color fundus photography, optical coherence tomography (OCT), fluorescein angiography (FA), indocyanine green angiography (ICGA), and OCT angiography to determine whether an MNV was present. Patients whose SCT was difficult to measure because of a submacular hematoma were excluded. Patients with other macular diseases secondary to the MNV such as high myopia, angioid streaks, and uveitis were also excluded. All patients underwent FA/ICGA with a confocal scanning laser ophthalmoscope (HRA2; Heidelberg Engineering Inc., Germany). Swept-source OCT with a 1050 nm wavelength light source (DRI- OCT; Topcon, Japan) was performed to measure the SCT and to determine the presence of a dry macula. Horizontal and vertical scans were routinely performed, and volume scans were performed when exudation was suspected at other areas.

### Conventional classification

We classified the patients as having one of three conventional subtypes of MNV secondary to AMD: PCV, typical type 1/type 2 MNV nAMD, and RAP (type 3 MNV). These MNVs secondary to AMD were characterized by exudative changes resulting from the presence of MNV as detected by fluorescein angiography and ICGA. All angiograms were evaluated individually by two of the four retina specialists (AK, RM, IM, TH) based on fundoscopic and angiographic findings, with the final diagnosis made by the other in case of discrepancy.

The diagnostic criteria for PCV were based on the presence of polypoidal lesions in the ICGA images. The diagnostic criteria for types 1, 2, and 3 MNV were based on the fundus OCT, FA, and ICGA findings^1^. Type 1 MNV in the FA images showed ill-defined lesions with multipunctate leakages. ICGA may help in viewing some of the vascular structure but often just shows late staining as a plaque. Type 2 in FA shows early, well-defined hyperfluorescence and the late leakage. Unambiguous cases of type 3 MNV were those with subretinal, intraretinal, or preretinal hemorrhages or retinal edema, and retina–retina anastomosis or retina–choroid anastomosis.

### Evaluation of choroidal images and reclassification

Choroidal vessels were evaluated in the ICGA images (HRA2, Heidelberg) or in the choroidal segmentation of *en face* OCT images (Elite 9000, Zeiss). The segmentation boundaries were adjusted to be at one-half of the SCT with a 30-mm width for the analysis of the medium and large of choroidal vessels in the *en face* OCT images. A pachyvessel was defined as a dilated choroidal vessel located in Haller layer. The large choroidal vessel compressed the inner layer of the choroid, and a PNV was defined as one in which a MNV was detected just above or contiguous to a pachyvessel. This definition of PNV did not use the choroidal thickness as a criterion. Pachyvessel and vertical asymmetry of the medium and large of choroidal vessels were evaluated independently by two co-authors (AK and IM).

Types 1, 2, and 3 MNVs were reclassified according to whether PNV was present. PCV was also classified as PNV or PCV depending on the presence or absence of polypoidal lesions and abnormal vascular network overlying the pachyvessels in the ICGA images.

The choroidal segmentation in the ICGA or *en face* OCT images was used to determine whether there was vertical asymmetry of the medium or large choroidal vessels. The SCT and vertical asymmetry of the eyes with PNV were compared to the eyes with non-PNV (without pachyvessels).

### Other fundus findings

As in the Age-Related Eye Disease Study, the fundus photographs at the initial examination were used to determine the presence of large drusen (size, ≥ 125 μm) extending around the lesion. This did not include pachydrusen involving dilated medium and large size choroidal vessels that are less clustered and more clearly demarcated than common drusen^37^. The presence of drusen was determined in the non-PNV eyes.

### Treatment responses

After diagnosis, the patients were treated with 3 consecutive monthly intravitreal injections of aflibercept or ranibizumab followed by the treat and extend (TAE) regimen. Patients received intravitreal injections using a 1-month interval adjustments to a maximum of 3 months for the remaining 2 years. If a dry macula was observed during the TAE adjustments, the interval to the next visit was extended by one additional month of the last interval. A dry macula was defined as the absence of subretinal and intraretinal spaces in the OCT images, and it was considered to be an indication of an absence of persistent and/or recurrent MNV activity. The interval was shortened by one month of the last interval if any of the following conditions were observed: (1) worsening of subretinal fluid, presence of intraretinal fluid or both in the OCT images; (2) new macular hemorrhage; (3) expanding pigment epithelial detachment; or (4) decreased visual acuity in the presence of retinal fluid in the OCT images. Our TAE regimen allowed us to shorten and extend the treatment intervals even after a 3- and/or 1-month treatment interval was reached. The minimum injection interval was 1 month and the maximum was 3 months. Our TAE regimen was based on the results of an earlier study^38^.

Forty-six eyes with PNV and non-PNV that were followed for at least 2 years were evaluated for the rate of dry maculae immediately after the loading phase, the number of treatments, and the final treatment interval at 2 years. The changes in the choroidal morphology were also evaluated.

### Statistical analyses

Statistical analyses were performed using the Mann–Whitney U-test or chi-square test. A value of *P* < 0.05 was considered to indicate a statistically significant different.

## Supplementary Information

Supplementary Figure S1.

## Data Availability

The datasets generated during and/or analyzed during the current study are available from the corresponding author on reasonable request.
