# Supplementary material for: Pachychoroid neovasculopathy has clinical properties that differ from conventional neovascular age-related macular degeneration
Source: Sci Rep. 2023 May 6;13:7379. doi: 10.1038/s41598-023-33936-z (PMC10164122; doi:10.1038/s41598-023-33936-z)
Supplement: Supplementary file 1 — Supplementary Figure S1. [file 41598_2023_33936_MOESM1_ESM.pdf]

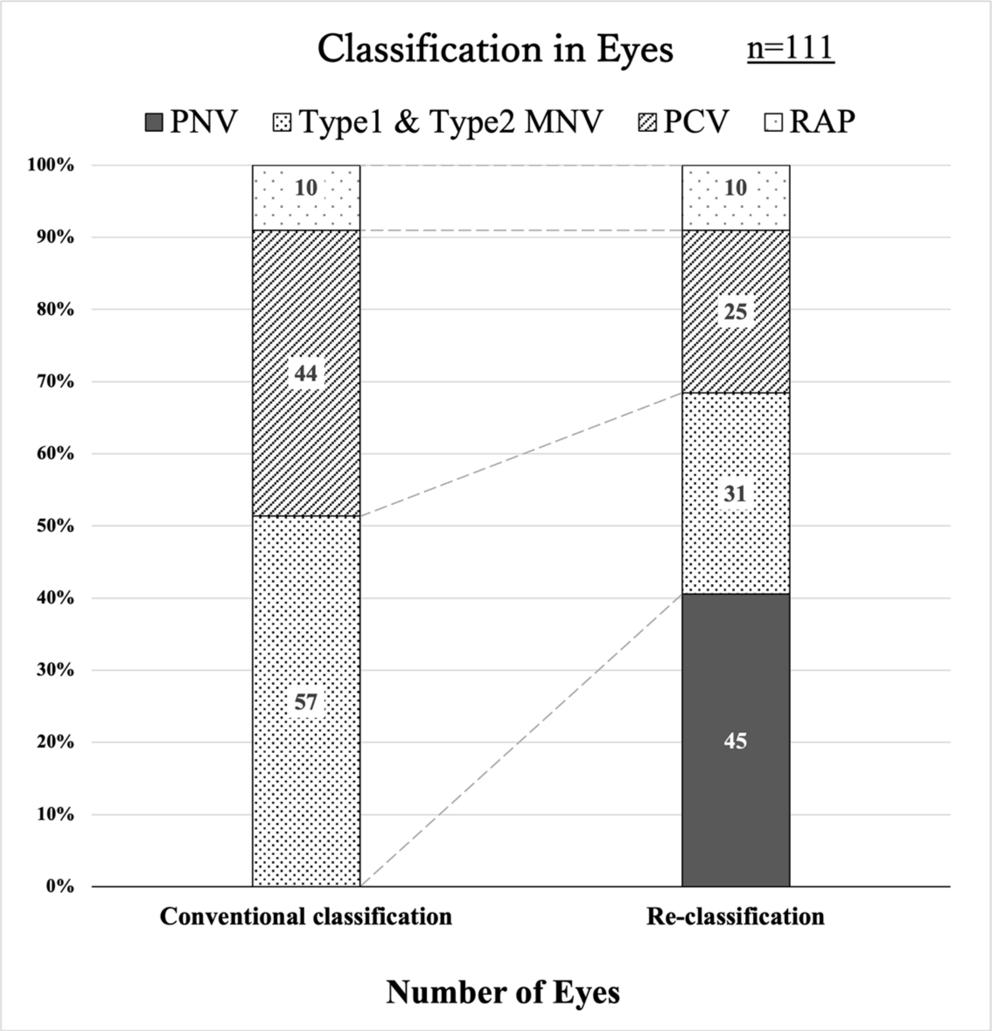


**Supplementary Figure S1.**

Conventional classification and re-classification in Eyes with MNV secondary to age-related macular degeneration and including PNV.

PNV, pachychoroid neovasculopathy; MNV, macular neovascularization; PCV, polypoidal choroidal vasculopathy; RAP, retinal angiomatous proliferation
